# Systematic video analysis of ankle sprain injuries in elite male football (soccer): Injury mechanisms, situational patterns, biomechanics and neurocognitive errors study: A study on 140 consecutive players

**DOI:** 10.1002/ksa.70049

**Published:** 2025-09-09

**Authors:** Matthew Buckthorpe, Evert Verhagen, Pieter D'Hooghe, Leonardo Osti, Stefano Di Paolo, Francesco Della Villa

**Affiliations:** ^1^ Faculty of Sport, Technology and Health Sciences St Mary's University London UK; ^2^ Education and Research Department, Isokinetic Medical Group FIFA Medical Centre of Excellence Bologna Italy; ^3^ Isokinetic Medical Group FIFA Medical Centre of Excellence London UK; ^4^ Department of Public and Occupational Health, Amsterdam UMC Amsterdam Collaboration for Health and Safety in Sports Amsterdam The Netherlands; ^5^ Department of Orthopaedic Surgery Aspetar Hospital Doha Qatar; ^6^ 2nd Orthopedic and Traumatologic Clinic IRCCS Istituto Ortopedico Rizzoli Bologna Italy

**Keywords:** ankle sprain injury, biomechanics, injury mechanism, injury prevention

## Abstract

**Purpose:**

To describe the mechanisms, situational patterns, biomechanics and neurocognitive errors related ankle sprain injuries of professional male football players during match play.

**Methods:**

There were 166 consecutive ankle sprain injuries identified occurring during official matches in players of top European football leagues. One hundred and forty (84%) injury videos were analysed for mechanism and situational pattern, with biomechanics on 20 players. Neurocognitive errors were investigated for all noncontact injuries. Three independent reviewers evaluated each video. Ankle sprain injury epidemiology—month, timing within the match and pitch location at the time of injury and time‐loss according to sprain type was also documented.

**Results:**

More injuries occurred in offensive (*n* = 89, 64%) than defensive (*n* = 51, 36%) situations (*p* < 0.001). Seventy (50%) direct contact, 42 (30%) indirect contact and 28 (20%) noncontact injuries were categorised. There were 67 (48%) inversion, 30 (21%) high ankle, 25 (17%) eversion, 6 with combination of high ankle and eversion (4%) and 12 (9%) unsure injuries. Four main situational patterns were described: (i) being tackled (*n* = 59, 42%); (ii) tackling/pressing (*n* = 34, 24%); (iii) landing from a jump (*n* = 16, 11%) and (iv) sliding (*n* = 7, 5%). Inversion injuries were associated with internal rotation, while high ankle injuries typically involved toe contact with the ground, slight plantar flexion and foot eversion. A neurocognitive error was documented 59% of noncontact injuries. A similar number of injuries occurred during the 1st (*n* = 71, 51%) and 2nd (*n* = 69, 49%) half (*p* > 0.05).

**Discussion:**

Half of ankle sprain injuries occurred after direct contact, 3 in 10 after indirect contact and only 2 in 10 without contact. Injury prevention practices should consider mechanical perturbation, playing situation and neurocognitive factors when designing programmes.

**Level of Evidence:**

Level IV.

**Abbreviations**
ACL: anterior cruciate ligament
DC: direct contact
IC: initial contact
IF: injury frame
InC: indirect contact
NC: noncontact
NY: New York
US: United States
USA: United States of America

## INTRODUCTION

The ankle is the most commonly injured body part across sports [23]. In elite male football (soccer), ankle injuries are the third most common injury (13%) [18, 52], with ankle sprains in general accounting for more than two‐thirds of ankle injuries and lateral ankle sprains representing more and half of all ankle injuries [52]. Although often considered innocuous injuries [17], ankle sprains have a high recurrence rate [20, 21, 22] and are often compounded by the development of persistent injury‐associated symptoms, including chronic ankle instability [16, 28]. Thus, the prevention of ankle sprain injuries is essential.

In designing injury risk mitigation programs, it is crucial to understand the injury epidemiology and aetiology [4, 49]. A key aspect of injury aetiology is establishing the contact mechanisms and context (situational patterns) where ankle sprains occur. Most of our understanding of ankle sprain injury mechanisms is based on recall, which is prone to bias [35]. Video analysis, despite not being the gold standard method of investigating the kinematics of injuries [31], is a valid tool [36] for studying the context (e.g., mechanisms and playing situations) of injuries. Video analysis has been used extensively in anterior cruciate ligament (ACL) injuries [8, 11, 40, 53], as well as other injuries in football, such as medial collateral ligament [9], Achilles tendon rupture [12] and muscle injuries [13]. One video analysis study was performed on ankle injuries in professional football more than 20 years ago, with a small sample size and no details of injury biomechanics [2]. More recent research included ankle injuries as part of a wider video analysis study of football injuries (66 injury videos) [30]. Still, it again did not consider the biomechanics of the injury or provide detailed information on situational patterns or injury epidemiology. Thus, there needs to be more understanding of the situations and biomechanics of ankle injuries in football. Some research has documented the injury mechanisms and biomechanics of lateral ankle sprains via video analysis in other sports [3, 22], with several case studies detailing biomechanics of ankle sprains during accidental injury during lab‐based investigations [24, 44]. While the latter offers high‐quality data, they lack ecological validity (e.g., football playing context). Given the unique nature of football, which involves player‐to‐player contact, grass/artificial turf as opposed to indoor surface, ball kicking and allowance of sliding tackling and contact scenarios, they likely possess different mechanisms and playing situations from many other sports. Furthermore, there have been many changes in football, including ankle preventive approaches, rules around tackling, match exposure and so on, highlighting a need to address this question regarding modern football ankle injuries. Finally, there is growing attention to the importance of neurocognitive errors in joint‐related noncontact injuries in football players [8, 26] which has not previously been considered for ankle sprains.

This study used video analysis to study the contact mechanisms, situational patterns, biomechanics (kinematics) and neurocognitive errors at the time of ankle injuries in professional male football players during competitive matches. A further purpose was to document the distribution of ankle sprain injuries according to sprain type (e.g., inversion, eversion and high ankle) and associated time loss as well as injuries across the match, season and pitch location. In studying how ankle sprain injuries occur in football, the study aims to support clinicians in diagnosing injuries and designing interventions to reduce injury incidence.

## MATERIALS AND METHODS

### Study design and injury identification

This study adopted a similar method to previously published studies on ACL [8, 11, 40], medial collateral ligament [9], Achilles tendon rupture [12] and severe muscle injuries [13] in football. The study utilised the ‘Quality Appraisal for Sports Injury Video Analysis Studies (QA‐SIVAS) scale’ [29] and reported a score of 16/18 (excellent) (see Supporting Information S1: Table S1). To summarise, the authors adopted a cross‐sectional retrospective observational study design. The authors systematically searched online database resources across three seasons (from 2018/2019 to 2021/2022 until 05/2022) to identify ankle sprain injuries occurring during matches in players of top European football leagues. Each season and team rosters were extracted from online databases (legaseriea.it; legab.it) and single‐team websites. Each player was then searched on the publicly available media‐based platform Transfermarkt.de (Transfermarkt GmbH & Co. KG) to ascertain details of their injury history. This methodology has been validated for injury identification in professional football [37], with sufficient validity and accuracy of retrieved injury‐related data reported and used in recent studies on return to play after various severe pathologies in professional football [27, 39, 46]. The search was supplemented by examining further data sources that may have been missed, including national and local media. Injuries were included only when we could corroborate the injury with official team media reports. Only injuries involving ankle sprain injuries and no additional fractures were included.

### Video extraction

Matches videos were obtained from an online digital platform (wyscout.com, Wyscout spa, [Genova Italy]) (*n* = 140). Videos were then processed on a digital cloud (paninidigitalcloud.com) and downloaded to a personal computer. Match video processing was done with a cloud‐available tool (Digital Log, Digital Football Project S.r.l.). Each injury video was cut to approximately 12–15 s before and 3–5 s after the estimated injury frame to accurately evaluate the playing situation that preceded the injury and injury mechanisms. Additionally, all available television replays (in slow motion and from different angles) were added to the video.

### Video evaluation and defining an ankle sprain injury

Three reviewers (M.B., L.O. and F.D.V.) independently evaluated the videos using two predetermined checklists (Supporting Information S1: Tables S2 and S3). All reviewers are involved in sports medicine and orthopaedic rehabilitation practice (PhD, MD, MD) and/or have extensive experience in video analysis research. Each video was downloaded onto a personal computer, opened with the available online software Kinovea (KinoveaInk), and analysed through an evaluation flow.

Each reviewer evaluated the original video to define the injurious situation, either defensive or offensive, which was determined based on ball possession and specific playing situations. A series of views was then used to determine the injury mechanism and situational pattern. The injured side was established based on injury history information and video data. The dominant leg was defined as the preferred kicking leg, categorised as right or left, and whether the injury occurred to the dominant kicking leg was assessed. Leg loading was classified as being on the injured, uninjured, or both limbs. Subsequently, the intensity of action was determined based on estimated horizontal and vertical velocities (zero, low, moderate and high). Three categories of injury mechanism were used according to previous research: [9, 11, 42] (1) direct contact, defined as an external force directly applied to the injured ankle and thought to be responsible for the injury; (2) indirect contact, defined as an injury resulting from an external force applied to the football player, but not directly responsible for the injury; this includes contact to the foot and/or shin and, while responsible for perturbation, is determined not to be the direct cause of the injury; (3) noncontact, defined as an injury occurring without any contact (at the ankle or any other level) before or at injury frame. We used the term ‘situational pattern’ to determine the playing action and context of the injury. This was conducted for all injuries and was interpreted as being tackled, tackling, pressing, landing from a jump, regaining balance after kicking, sliding, offensive change of direction, running, ball kicking or other.

Following independent analysis, the reviewers met for 1 day to achieve consensus on all items regarding injury mechanisms and situational patterns and perform the biomechanical (kinematic) analysis (described below). Disagreements were resolved via consensus [11, 45, 53]. Before the meeting, the intraclass correlation index between the reviewers for the initial contact and injury frame was 0.99.

### Biomechanical analysis (kinematics)

Kinematic analysis was performed on noncontact and indirect contact injuries, like previous research [9, 11], when a sufficient quality frontal and/or sagittal view was available. The kinematic analysis was undertaken during the consensus meeting. The analysis estimated the intersegmental relationship and joint angles according to the frontal and sagittal plane alignment at initial contact and injury frame, focusing on the foot.

Sagittal plane angles (hip, knee and ankle flexion) and ankle and trunk tilt were evaluated using custom‐made software (Screen Editor, GPEM srl). The estimated joint positions of the remaining frontal (hip abduction/adduction; knee valgus/varus) and coronal planes (trunk and foot rotation) were categorised according to appearance. Foot strike was evaluated according to the previous methodology [11, 53] and after foot contact to the ground at initial contact and injury frame. Foot inversion/eversion angles were reported. The items that have been evaluated are listed in Supporting Information S1: Table S3.

### Neurocognitive analysis

We adopted the same neurocognitive analysis process as in previous work on ACL injuries [8, 26], with a view to explore the role of inhibitory control. Inhibitory control describes the ability to control attention, behaviour, thoughts, emotions or a combination of these to cancel strong internal predispositions or external temptations and instead act in a more appropriate way [15]. Like previous research [8, 26], we were interested in two aspects of inhibitory control: attentional inhibition and motor response inhibition [15, 26].

Attentional inhibition refers to the ability to resist interference from stimuli in the external environment [26]. For attentional inhibition, the players' selective attention was defined as focusing on a particular situation for some time [15]. An error in attentional inhibition was determined if the injured player shifted their selective attention (i.e., looking somewhere else) away from the relevant action, leading to injury to other nontask relevant stimuli, for which the player could not directly impact, such as the ball/environment, suggestive of attentional inhibition. We considered that loss of attentional focus/direction of visual attention may lead to spatial unawareness and altered neuromuscular control (e.g., altered muscle preactivation before landing) [26].

Second, we defined motor response inhibition as stopping unwanted and incorrect motor actions [8, 15, 26]. Motor‐response inhibitory control blocks behaviours and stops inappropriate automatic reactions, changing one response for a better, more thought‐out response adapted to the situation. As there is a delay between the presentation of a stimulus (e.g., opposing players deceiving action) and generating an appropriate reactive response [25], we, in line with previous research, operationalised the player had ∼450–1200 ms to change the motor response (e.g., react to a deceiving action of a player such as faking to go one direction and moving in another) [25, 26]. For this assessment, on the checklist, the reviewers also had to record if, during noncontact injuries, the opposing player performed a deceiving action during defensive situations (e.g., pressing or tackling injuries) indicative of poor inhibitory control.

### Seasonal, match and field distribution

For each available injury video, a list of data regarding the seasonal, match and field distribution was gathered through the systematic web revision and the analysis of the videos about the injured player's location on the pitch. We recorded: (1) the month of the ankle sprain injury, (2) the phase of the game when the ankle sprain injury occurred (minute and a half), (3) number of minutes played by the ankle sprain injured player considering substitutions and (4) pitch location according to previously published methodology [11, 12, 13]. Player location at the time of ankle sprain injury was gathered according to the pitch lines. The football pitch was divided into 11 zones according to Supporting Information S1: Tables S4 and S5 [11]. The pitch zone dimensions in square meters were calculated considering the official FIFA football field size (105 by 70 m) (see Supporting Information S1).

### Statistical analysis

Normal distribution of data was inspected through the Shapiro–Wilk test. Continuous variables have been presented as mean (±standard deviation) or median (range) as appropriate according to the variable's distribution. Discrete variables were presented as absolute numbers and percentages on the number of total observations. Since lay‐off time was nonnormally distributed (*p* < 0.05), the Kruskal–Wallis test was used to compare the means of days missed among direct contact, indirect contact and noncontact ankle injuries and high, eversion and inversion ankle sprains. The Mann–Whitney *U*‐test was used to compare each subgroup with Bonferroni correction for post hoc analysis. Partial eta‐squared and rank biserial correlation coefficients were reported alongside *p*‐values to measure effect size. The proportion test was used to explore possible differences in the temporal distribution of ankle injuries. An alpha less than 0.05 denoted statistical significance. Microsoft Excel 2016 (Microsoft) and SPSS (v26, IBM) were used for these analyses.

## RESULTS

**Figure 1:**
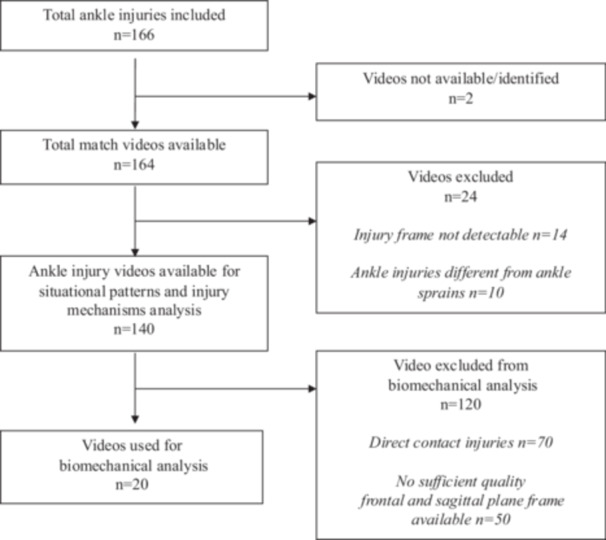
A detailed study flow, showing total ankle injuries included, the number of obtainable match videos, those match videos available for analysis of situational pattern and injury mechanisms and those videos of sufficient quality for biomechanical analysis.

One hundred and sixty‐six ankle sprain injuries were tracked and included. The injured players age was 25.7 ± 3.5 years. Injuries across leagues are distributed in Supporting Information S1: Table S6. Injuries occurred in two goalkeepers, 72 defenders, 41 midfielders and 51 forwards. There were 89 (54%) injuries to the right and 68 (41%) injuries to the left ankle (9 injuries were unidentifiable), with 76 (46%) injuries to the dominant kicking leg and 80 (48%) to the nonkicking leg. A detailed study flow is shown in Figure 1.

### Injury mechanism analysis

Video footage was available and identifiable for situational pattern and injury mechanism analysis in 140 players (84%). More injuries occurred in offensive (*n* = 89, 64%) than defensive (*n* = 51, 36%) (*p* < 0.001) situations. Most injuries (122 players; 87%) involved loading of the injured leg, with single limb loading on the ground frequently observed (109 players; 78%). Seventy (50%) direct contact, 42 (30%) indirect contact and 28 (20%) noncontact injuries were categorised. For indirect contact injuries, 22 (52%) of these involved contact to the foot (*n* = 12) and/or tibia (*n* = 10) before or at injury frame, while 20 (48%) involved contact to other body sites including the upper body (*n* = 14), contralateral leg (*n* = 4) and pelvis (*n* = 2).

Injuries according to sprain type were 67 (48%) inversion, 30 (21%) high ankle, 25 (18%) eversion, 6 with combination of high ankle and eversion (4%) and 12 (9%) unsure cases. A large proportion of injuries involved high or moderate horizontal speeds (102 cases, 73%), while few (*n* = 17, 12% of cases) involved high or moderate vertical speeds at initial contact (Table 1).

**Table 1:** Details of injury mechanism analysis of ankle sprain injuries assess video analysis according to a predetermined checklist (*n* = 140).

Variables	Results
Weather conditions	
Raining	No (*n* = 129), yes (*n* = 11)
Sunny	Night (*n* = 86), yes (*n* = 27), no (*n* = 27),
Playing phase before injury	Offensive (*n* = 89), defensive (*n* = 51)
Field location of the injury	
The long axis of the field	Defensive third (*n* = 43), midfield third (*n* = 50), offensive third (*n* = 47)
The short axis of the field	Left side corridor (*n* = 22), middle corridor (*n* = 87), right side corridor (*n* = 31)
Player contact preceding injury	Yes (*n* = 73), no (*n* = 67)
If contact, where?	Injured leg (*n* = 31), upper body (*n* = 30), uninjured leg (*n* = 7), pelvis (*n* = 5)
Contact at IC perceived relevant to injury?	Yes (*n* = 48), no (*n* = 25)
Player contact at IF	Yes (*n* = 87), no (*n* = 53)
If contact at IF, where?	Injured leg (*n* = 73), upper body (*n* = 12), uninjured leg (*n* = 2)
Contact at IF perceived relevant to injury?	Yes (*n* = 81), no (*n* = 6)
Injury mechanism (contact)	DC (*n* = 70), InD (*n* = 42), NC (*n* = 28)
Injury mechanism (sprain type)	Inversion (*n* = 67), (DC 30, InC 26, NC 11); high ankle (*n* = 30), (DC 12, InC 8, NC 10); eversion (*n* = 25), (DC 19, InC 4, NC 2); eversion/high ankle (*n* = 6) (DC 6); unsure (*n* = 12), (DC 3, InC 4, NC 5)
Number of feet on the ground at IF	One (*n* = 109), two (*n* = 29), unsure (*n* = 2)
Leg loading at IF	Injured leg (*n* = 122), unsure (*n* = 15), uninjured leg (*n* = 2), none (*n *= 1)
Horizontal speed	Zero (*n* = 3), low (*n* = 35), moderate (*n* = 64), high (*n* = 38)
Vertical speed	Zero (*n* = 114), low (*n* = 9), moderate (*n* = 14), high (*n* = 3)

*Note*: Eversion and high (*n* = 6). Abbreviations: DC, direct contact; IC, initial contact; IF, injury frame; InC, indirect contact; NC, noncontact.

### Situational patterns

**Figure 2:**
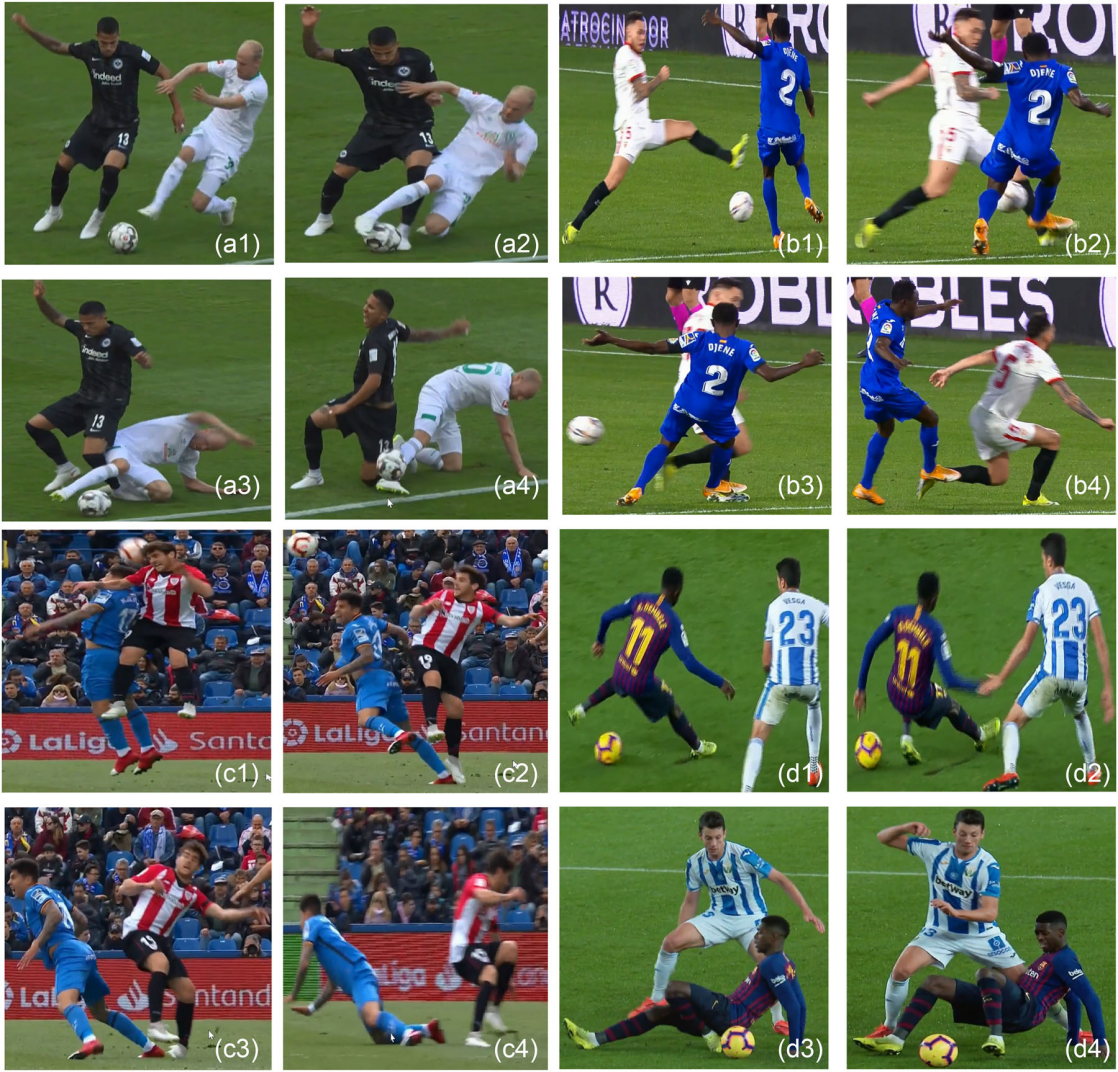
Visual representation of the main situational patterns. (a) *being tackled* (injured player in black jersey, left ankle sprain); pressed by opposing player, with attempted sliding tackle (a1), initial contact with the ground and player contact to the injured leg (a2), estimated injury frame (a3), loss of balance (a4). (b) *tackling* (injured player in blue kit, right ankle sprain); pressed by opposing player (b1), dual tackle for ball (b2), estimated injury frame (b3, moments after injury (b4). (c) *landing from a jump* (injured player in red and white jersey, left ankle sprain); aerial heading dual (c1), initial contact with opposing players foot on ground (c2), estimated injury frame (c3) regaining balance on uninjured side after ankle injury (c4). (d) *sliding* (injured player in blue and burgundy jersey, left high ankle sprain); foot plant and sliding on the uninjured side (d1), initial contact with the ground with toe (injured left side) (d2), estimated injury frame of left ankle (d3, moments after injury (d4).

We reported four main situational patterns: (i) tackled (*n* = 59, 42%); (ii) tackling/pressing (*n* = 34, 24%); (iii) landing from a jump (*n* = 16, 11%) and (iv) sliding (*n* = 7, 5%) (Figure 2) accounting for 83% of injuries. The other 24 (17%) injuries did not fall into one of these four patterns and involved regaining balance after kicking (*n* = 5), collision with another player (*n* = 5), offensive change of direction (*n* = 4), blow to ankle or toe by the ball (*n* = 3), kicking/shooting (stance leg injured) (*n* = 2), reaching for the ball (stance leg injured) (*n* = 2), running (*n* = 2) and sidestepping (*n* = 1). Further details, including a breakdown of situational patterns and mechanisms, are detailed in Table 2.

**Table 2:** Ankle sprain injuries assessed using video analysis according to situational pattern, their playing situation, contact mechanisms of injury and sprain type mechanism (*n* = 140 cases).

Situational pattern	Playing situation	Contact mechanism	Ankle sprain‐type mechanism
Tackled, *n* = 59 (42%) − Inside blow, *n* = 21 − Outside blow, *n* = 33 − Front blow, *n* = 1 − Unsure, *n* = 4	Offensive, *n* = 59	DC, *n* = 57; InD, *n* = 2	Inversion, *n *= 21; high ankle, *n* = 12; eversion, *n* = 17; eversion/high, *n* = 6, unsure, *n* = 3
Pressing/tackling, *n* = 34 (24%) − Tackling, *n* = 28 − Pressing, *n* = 6	Defensive, *n* = 34	DC, *n* = 11; InC, *n* = 16; NC, *n* = 7	Inversion, *n* = 23; high ankle, *n* = 8; eversion, *n* = 3
Landing from a jump, *n* = 16 (11%)	Defensive, *n* = 6; offensive, *n* = 10	InD, *n* = 12; NC *n* = 4	Inversion, *n* = 8; eversion, *n* = 3; unsure, *n* = 5
Sliding, *n *= 7 (5%)	Defensive, *n* = 3; offensive, *n* = 4	InD, *n* = 2; NC, *n* = 5	High ankle, *n* = 7
Other, *n* = 24 (17%)	Defensive, *n* = 8 offensive, *n* = 16	DC, *n* = 3; InD, *n* = 10; NC, *n* = 11	Inversion, *n* = 15; high ankle, *n* = 3; eversion, *n* = 2; unsure, *n* = 4

Abbreviations: DC, direct contact; InC, indirect contact; NC, noncontact.

### Biomechanical analysis

Biomechanical analysis was possible in 20 noncontact and indirect contact ankle sprain cases. These included 12 inversion and eight high ankle injuries. All angle data are reported as median values. Key biomechanical features for inversion injuries on the sagittal plane at initial contact include a slightly plantarflexed ankle (−7.5°), predominantly with a toe (50%) or midfoot (33%) foot strike appearance. On the frontal plane at initial contact, the ankle was in inversion (12.5°) and generally in internal rotation (58%). At estimated injury frame, on the sagittal plane, there was a minimal increase in plantarflexion (−10°, −2.5° from initial contact), with nearly all injuries involving flat foot strike appearance (92%). On the frontal plane, there was a large increase in ankle inversion (70°, +57.5° from initial contact), with the foot always internally rotated (100%).

**Figure 3:**
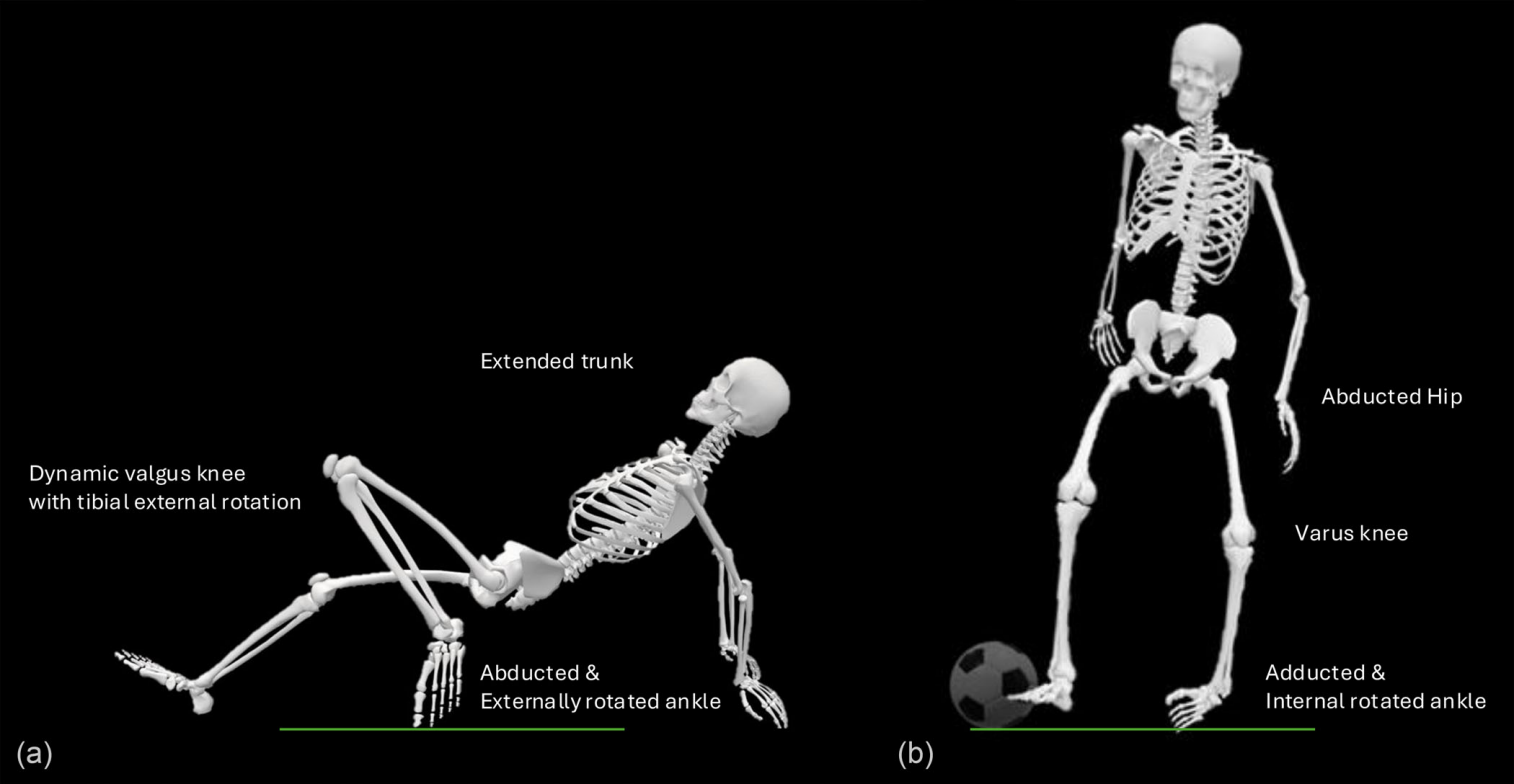
Frequently observed kinematics for noncontact and indirect contact ankle sprain injuries for (a) High ankle injuries and (b) Inversion injuries.

Key biomechanical features for high ankle sprain injuries on the sagittal plane at initial contact involved a plantarflexed ankle (−12.5°), with a heel (38%), flat (37%) or toe (25%) foot strike appearance. On the frontal plane at initial contact, the ankle was in inversion (12.5°) and generally in internal rotation (58%). The foot was generally neutral (50%) but also externally (25%) and internally rotated (25%). At estimated injury frame, on the sagittal plane, the knee was in full flexion (160°, +100° from initial contact), with minimally reduced plantarflexion (−10°, +2.5° from initial contact) and all with toe foot strike appearance. On the frontal plane, inversion was not identifiable, but the foot was always in external rotation (100%). See Table 3 for more details and Figure 3 for injury frame's most frequent intersegmental positioning.

**Table 3:** Sagittal plane metrics of noncontact or indirect contact ankle sprain injuries (*n* = 20).

Variables	Inversion (*n* = 12)		High (*n* = 8)	
	IC	IF	IC	IF
Flexion angle (°) (+ flex., − ext.)				
Trunk	10 (−15, 50)	17.5 (−5, 45)	−10 (−25, 35)	−17.5 (−85, 35)
Hip	42.5 (0, 70)	40 (10, 70)	67.5 (40, 115)	90 (50, 115)
Knee	30 (15, 95)	47.5 (15, 65)	60 (10, 115)	160 (100, 170)
Ankle (−Pflex., +Dflex.)	−7.5 (−65, 25)	−10 (−35, 35)	−12.5 (−45, 30)	−10 (−35, 20)
Foot strike appearance				
Heel	2 (17%)	0 (0%)	3 (38%)	0 (0%)
Flat	4 (33%)	11 (92%)	3 (37%)	0 (0%)
Toe	6 (50%)	1 (8%)	2 (25%)	8 (100%)
Trunk tilt (+ ipsi, −contra.)	0 (−15, 45)	0 (−15, 40)	2.5 (−40, 45)	25 (−5, 50)
Trunk rotation				
Towards injured	2 (17%)	1 (8%)	4 (50%)	3 (38%)
Neutral	6 (50%)	5 (42%)	3 (38%)	4 (50%)
Towards uninjured	4 (33%)	6 (50%)	1 (12%)	1 (12%)
Frontal plane hip alignment				
Abduction	9 (75%)	12 (100%)	5 (63%)	8 (100%)
Neutral	3 (25%)	0 (0%)	1 (12%)	0 (0%)
Adduction	0 (0%)	0 0%)	2 (25%)	0 (0%)
Frontal plane knee alignment				
Valgus	3 (25%)	2 (17%)	3 (38%)	5 (63%)
Neutral	6 (50%)	4 (33%)	3 (37%)	3 (37%)
Varus	3 (25%)	6 (50%	2 (25%)	0 (0%)
Ankle tilt (+ inv, − ev)	12.5 (−5, 45)	70 (5, 85)	—	—
Foot position (rotation)				
External	3 (25%)	0 (0%)	2 (25%)	8 (100%)
Neutral	2 (17%)	0 (0%)	4 (50%)	0 (0%)
Internal	7 (58%)	12 (100%)	2 (25%)	0 (0%)

*Note*: Frontal and transverse plane metrics of noncontact or indirect contact ankle sprain injuries reported across high ankle (*n* = 8) and inversion injuries (*n* = 12).

Abbreviations: contra., contralateral; Dflex., dorsi‐flexion; ev, eversion; ext. extension; Flex, flexion; IC, initial contact; IF, injury frame; inv, inversion; ipsi, ipsilateral; Pflex., plantarflexion.

### Neurocognitive factors

Of 27 noncontact injuries, 14 (59%) were deemed to involve a neurocognitive error, with 10 (71%) involving an attentional error and four (29%) injuries involving motor inhibitory response. The attentional errors (*n* = 10) were present in landing from a jump (*n* = 4), regaining balance after kicking (*n* = 3), offensive COD, reaching and side stepping. Of those classified as motor response inhibition, all four involved a deceiving action from the opposing player during pressing injuries.

### Lay‐off times

Median lay‐off time was 36.0 [22.5; 72.8] days. Sixty‐three (38%) injuries were classified as moderate (14–28 days) and 103 (62%) as severe (>28 days). There was no difference in lay‐off times between direct contact (39.5 [23.0; 80.5] days), indirect contact (38.0 [28.0; 72.0] days) and noncontact (31.0 [19.0; 54.0] days) (*p* > 0.05). There was a significant difference in time‐loss according to the sprain type mechanism (*p* = 0.014, *η*²_*p*_ = 0.069), in which high ankle sprains had longer time‐loss than inversion ankle sprains (52.5 [26.5; 88.5] days vs. 31.0 [18.5; 50.5] days, *p* = 0.011), with eversion ankle sprains (39.0 [28.0; 71.0] days) being no different from high ankle and inversion sprains (*p* > 0.05).

### Seasonal, match and field distribution

**Figure 4:**
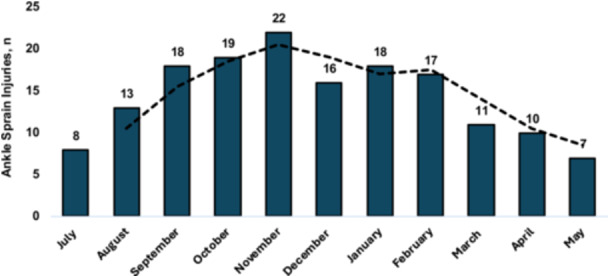
Distribution of ankle sprain injuries (*n* = 159) throughout the football season according to month of the year. The trend line displays the 2‐month rolling average.

Data for seasonal (*n* = 159), match timing (*n* = 140) and field distribution (*n* = 150) were available. The seasonal distribution demonstrated a shallow ‘n’ shape, in which the peak occurred in November (*n* = 22, 14%) but with high and similar incidence from September to February (range, 16–22) (Figure 4).

**Figure 5:**
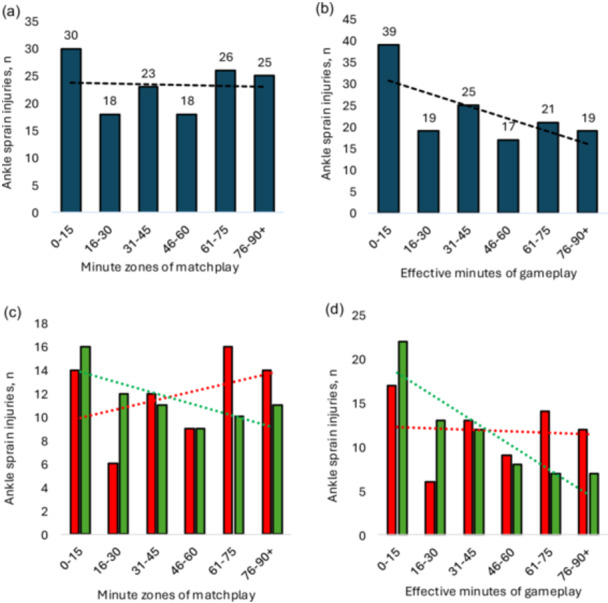
Distribution of ankle sprain injuries throughout the match according to match minutes and specific time zone/period (all injuries *n* = 140) (a) and minutes of effective playing time (all injuries *n* = 140) (b). Dashed lines represent a linear trendline. (c, d) The distribution of ankle injuries across the match according to match minute (c) and effective minutes played (d), according to the contact mechanism. Direct contact injuries (*n* = 70) are presented in red, and indirect and noncontact injuries (*n* = 70) are presented in green. Dashed lines are in the colour of the associated columns and represent the linear trend.

**Figure 6:**
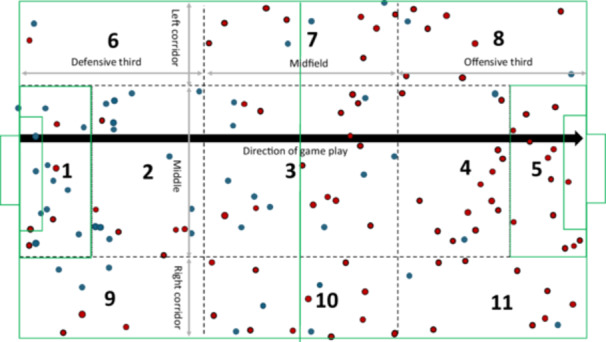
Distribution of ankle sprain injuries (*n* = 140) on the football pitch according to a modification of a previously published division of the football field. The grey arrows indicate the divisions along the length and width of the field, and the dashed black lines mark the boundaries of the 11 zones. The blue dots represent defensive injuries, and the red dots represent offensive injuries. The bold black line and arrow represent the direction of gameplay.

A similar number of injuries occurred during the first (*n* = 71, 51%) and second (*n* = 69, 49%) half (*p* > 0.05) (Figure 5). When considering the minutes played, correcting for substitutions, 28% (*n* = 39) of ankle sprain injuries occurred in the first 15 min (Figure 5). When breaking down injuries according to contact mechanism, a similar number of injuries were apparent for direct contact injuries in the first (*n* = 32, 45%) and second (*n* = 39, 55%) half (*p* > 0.05). Likewise, a similar number of injuries were apparent for indirect and noncontact injuries in the first (*n* = 39, 57%) and second halves (*n* = 30, 44%) half (*p* > 0.05) (Figure 5). Figure 6 and Supporting Information S1: Tables S3 and S4 describe injuries according to pitch location.

## DISCUSSION

The most important findings of this study are that most ankle sprain injuries in elite male football are due to some form of contact. Half are ankle sprain injuries were direct contact, 3 in 10 indirect and 2 in 10 purely noncontact. This work suggests four main situational patterns: (1) being tackled, (2) tackling/pressing, (3) landing from jump and (4) sliding. Inversion injuries were more common and involved consistent inversion and internal rotation biomechanics. High ankle injuries were common, had greater time‐loss and involved consistent biomechanics. In those noncontact ankle sprains, a neurocognitive error was apparent in three out of five injuries. A similar number of injuries occurred across halves.

### Injury mechanisms

The results of this study support previous research on football, which found direct contact ankle sprain injuries to be most prevalent [2, 30, 54]. Only one in five were noncontact, while three in 10 were indirect contact. While less frequent, the noncontact and indirect contact injuries highlight opportunities for injury risk mitigation through targeted injury prevention/neuromuscular training. The noncontact proportion in previous research ranged from 8% to 42% [2, 30, 52, 54]. Those video analysis studies have documented 8%–12% of noncontact injuries [2, 30]. Our work supports indirect contact as a major injury mechanism (30%), like previous research (29%) [30]. Half of the indirect contact injuries involved contact to the foot and/or tibia at or before initial contact. The rationale for classifying these injuries as indirect contact, as opposed to direct contact, like some research [3], was that they were not deemed directly responsible for the injury but instead led to foot ‘perturbation’, with the player's momentum resulting in the required forces to elicit injury. It is plausible that a stiffer ankle (increased muscle preactivation or passive support such as brace/taping) at the point of contact may not have perturbed to the same extent [14, 55] and thus may have been preventable.

### Sprain‐type and time‐loss

Half of the injuries were inversion injuries (48%), with the remainder being high ankle (22%), eversion (17%) or a combination of high ankle and eversion (4%). Walden et al. [52] reported that 76% of injuries affected lateral ligaments, with medial (10%) and high ankle (5%) sprains being less common. The greater proportion of high ankle injuries in our study versus previous research could result from a greater understanding of the aetiology and diagnosis of high ankle sprains and our sample size involving more severe injuries.

The lay‐off times in this study were longer than previously reported in football (36 vs. 13–25 days) [18, 19, 28, 30, 52], with a higher proportion of severe injuries (62% vs. 13%) [18, 51]. Discrepancies in injury reporting methods likely explain this difference. The similar time loss for inversion and eversion injuries and longer time loss for high ankle injuries correspond to previous research [18, 52].

### Situational patterns

This study identified four main situational patterns for ankle sprain injuries: (i) being tackled, (ii) tackling/pressing, (iii) landing from a jump and (iv) sliding. If considering the ‘tackle’ as an inciting injury (including being tackled and tackling), collectively, they explained 62% of all injuries. This supports previous research and Andersen et al.'s [2] statements that challenging ball possession is a high risk for ankle sprain injuries in football. Nearly two‐thirds of injuries occurred in offensive situations, likely due to being tackled as the predominant pattern. Only Andersen et al. [2] have previously suggested situational patterns for ankle sprain injuries in football. This study found a similar proportion of injuries while being tackled (42% vs. 38%), tackling (20% vs. 15%) and landing (11% vs. 8%) to their work. Andersen et al. [2] classified kicking or shooting the ball as a main pattern (15%), unlike this study (*n* = 2, 1%). This may relate to changes in technique/skill and differences in footwear and football (e.g., lighter football versus 2003) over those 20 years. The sliding mechanism is novel (distinct mechanisms and different kinematics, discussed below) and implicated in indirect and noncontact high ankle injuries. Further research to investigate its biomechanics and potential preventative strategies is warranted. This study reported few running injuries (1%) compared with Andersen et al. [2] (15%).

### Biomechanics (kinematics)

The lateral ankle sprain injury has historically been described as a plantarflexion‐inversion injury [2]. This study confirms that inversion injuries (considered lateral ankle sprains) result from inversion with internal rotation, supported by much research [24, 34, 41, 44, 47]. The degree of inversion reported in our study corresponds to published data (70° vs. 67.5°) [40]. The lack of plantarflexion forming an essential part of the injury sequence is supported by previous video analysis research in court‐based sports [3, 22, 24, 44] and quantitative analyses from laboratory events and detailed 3‐dimensional assessment [24, 34, 44].

More research on the biomechanics of high ankle sprains needs to be published, with most research describing injury kinematics via player recall and medical staff questionnaires [38]. This study showed a consistent pattern during sliding movements, resulting in toe contact with the ground, large knee flexion and external foot rotation. These findings support evidence that these injuries occur due to external foot rotation [10]. During the external rotation of the foot, the fibula rotates externally and translates posteriorly [5]. The talus externally rotates, producing a widening of the ankle joint mortise, thought to result in a sprain of the anterior inferior and posterior inferior tibiofibular ligaments. Previous research has reported forced dorsiflexion is a key mechanism of these injuries [10, 38], which this study questions. This study noted a 10° median plantarflexion, although plantarflexion was not consistent with a range from 35° plantarflexion to 20° dorsiflexion across the eight injuries. Further research to delineate this via model‐based image‐matching techniques is warranted.

### Neurocognition

It was reported that a ‘neurocognitive error’ occurred in three out of every five (59%) noncontact ankle sprains. This finding is novel and suggests neurocognitive errors may have contributed to the events leading to a noncontact ankle sprain injury, like previous research on noncontact ACL injuries [8, 26]. Of the neurocognitive errors, most were attentional errors (11/14, 79%), which differs from noncontact ACL injuries where motor response inhibition was more common [8, 26]. This externally directed attention away from the movement task (e.g., impact landing) may have resulted in attention being taken away from temporospatial awareness of the player's movement possibly compromising motor control and leading to ankle sprain injury. Of note, all noncontact landing from jump injuries involved an attentional error, in which the players attention was focused external to the ground prior to ground contact.

Only four of the 27 noncontact ankle sprains involved a neurocognitive error in response to a deceiving action by the opposing player, classified here and elsewhere as ‘motor response inhibition’ [8, 26]. These were all pressing inversion injuries. When an opposing player performs a deceiving action, the defender must react quickly, inhibit an already initiated response and plan and execute a new movement within this short time window (~250 ms) [26]. Ankle sprain injuries are thought to occur within 60–110 ms after ground contact [24, 44], which is like the evertor muscle stretch‐reflex response (60–120 ms) [14, 33, 48]. Thus, optimal feedforward motor patterns with appropriate muscle preactivation to develop tension and stabilise the joint before initial contact is essential in ankle injury prevention [47, 55]. It is possible that the altered biomechanics at the time of injury were a result of a fast‐initiated reactive movement task, with errors in response to a deceiving action of the opposing attacking player. This further proves that deceiving an opposing player's actions during pressing situations is an at‐risk situation for lower limb noncontact joint injuries.

### Seasonal, positional, match and field distribution

An inverted ‘u’ shaped distribution of injuries was reported across the season. The lower injuries in the May, July and August months are likely due to match exposure, which this study did not document. Previously, Walden et al. [52] documented a shallow ‘m’ shape distribution, but controlled for exposure.

Fatigue is not a dominant main risk factor for ankle sprain injuries, as a similar proportion of injuries occurred across halves. However, it still may be implicated in ankle sprains [51] as part of a complex web of determinants [6]. Walden et al. [52] on a large sample of ankle injuries, reported fewer injuries in the first 15 min of the match, with a similar number across the other 15‐min periods. This work contradicts this with a similar or higher number of injuries in the first 15 min of match play. When correcting for substitutions, the first 15 min of exposure accounted for 28% of injuries. This may suggest a proportion of injuries are associated with insufficient player readiness, and/or more intense engagements at the onset of match play.

More injuries were documented in defenders (43%) than midfielders (25%) and forwards (31%) in this study. Andersen et al. [2] reported in a small cohort (*n* = 25 outfield players) that more than half (58%) of injuries occurred in midfield players, 16% in strikers and 28% in defenders. Differences may relate to a low number of players and video availability in their study, as well as a change in football profile over the last 20 years, rule changes, and more varied formations (e.g., 4‐3‐3 vs. 4‐4‐2). This study did not quantify exposure, and the authors do not suggest this work highlights greater risk for defenders for ankle injuries, just that this sample of player involved more injuries in defenders versus previous research. Further research to assess ankle injury risk between playing positions, controlling for exposure if warranted.

### Practical implications

Understanding injury mechanisms is key to designing effective injury risk mitigation practices [4, 49]. Most ankle injuries involve contact, which suggests they are largely not preventable. One in five ankle injuries was noncontact, and three in 10 were from indirect contact, which should be the focus of injury risk mitigation interventions. In elite male football, we suggest most of the focus on ankle injury risk mitigation be targeted at previously injured players, as well as incorporated as part of the rehabilitation and return to play process, focusing on secondary injury risk mitigation. Current practices for ankle injury prevention typically focus on balance board/proprioceptive training, which has shown a positive effect on ankle injury risk [1], specifically secondary injury risk [43, 50]. However, this type of training lacks specificity to the nature of the injury. Here and elsewhere, ankle injuries in football have been shown to involve complex environmental demands (situational patterns) and/or physical contact (three in 10 being indirect contact). The short time (60–120 ms) for joint re‐stabilisation following neuro‐ or mechanical‐perturbation [14, 24, 33, 44, 48] reiterates the importance optimal feedforward motor patterns with appropriate muscle preactivation to develop joint stiffness tension prior to ground or player contact. This contrasts with the feedback‐driven motor strategies, indicative of balance training. Designing prevention programs incorporating perturbation training and mimicking game‐like scenarios such as landing and rapid direction changes may mitigate injury risk. Those programs employing landing and plyometric training alongside balance training have shown a reduced risk of ankle injuries [1], potentially due to enhanced muscle preactivation. Return to sport practices should recognise the complexity of the injury context and ensure players are exposed to movements under more game like context (e.g., reactive change of direction, pressing actions with distractions, landing actions with complex environmental demands and perturbation) before commencing the return to play process (e.g., return to training and subsequent competitive match play) [7].

### Methodological considerations and future research

Most previous research on ankle sprain injury mechanisms has relied on player recall or observations with questionnaires, which are prone to recall bias. This study addressed this issue by using video analysis of actual injuries. The consecutive nature of the injuries analysed and the consistent biomechanical/kinematic analysis of three independent raters (using measurement tools) are strengths. Furthermore, this is the only video analysis study specifically on football for ankle injuries in the last 20 years [2], with a large sample size (140 injuries). However, the study does have some limitations. This work relied on ‘Transfermarkt’ and media sources for injury data, which differs from the gold standard method, being prospective studies with frequent contact with teams [52] supported by exposure data. This may skew and limit the epidemiological findings, particularly related to lay‐off times. Furthermore, excluding training injuries could interfere with the overall presentation of ankle sprain injuries in professional football. Further research on training injuries, including video analysis of events, is warranted. This study could not distinguish between grades of ankle injury or parts of the ankle injured. Video analysis is a valid [36] and consistently adopted approach for analysing actual injuries in the sporting context and is utilised in similar studies [2, 3, 10]. However, model‐based image‐matching techniques are considered the gold standard method of injury analysis [22, 24, 31, 32], and follow‐up research using model‐based image‐matching techniques on football injuries is warranted. There were few indirect and noncontact injuries for full biomechanical analysis (*n* = 20), but this sample size is still relatively large versus most previous research, which typically involved case studies or small cohorts [10, 22, 24, 44, 47]. The results may not generalise to females and players of lower competitive levels and ages.

## CONCLUSION

Ankle injuries are generally contact injuries, with only one in five occurring in noncontact situations, and as such injury risk mitigation strategies may be minimally effective. They are more common in offensive situations and occur during four main situational patterns: being tackled, tackling/pressing, landing from jump, and sliding. Half of the injuries were inversion sprains, occurring with inversion and internal rotation, while high ankle sprains involved toe contact to the ground with foot eversion. Noncontact injuries generally involve a neurocognitive error, and so challenging neurocognition during injury prevention exercises is recommended. A similar number of injuries occurred across halves, but a greater proportion in the first 15 min of effective playing time, suggesting fatigue is not the main factor in their causation.

## AUTHOR CONTRIBUTIONS

Matthew Buckthorpe and Francesco Della Villa were responsible for the conception and design of the study. Leonardo Osti supported the collection of videos, injury data and video editing. Matthew Buckthorpe, Leonardo Osti and Francesco Della Villa, analysed and rated the video footage regarding injury mechanism and situational patterns. Matthew Buckthorpe analysed and interpreted the results. Matthew Buckthorpe wrote the first draft of the manuscript. All authors provided intellectual contribution to the writing and drafting of the manuscript. Matthew Buckthorpe and Francesco Della Villa were responsible for the overall content as guarantors.

## CONFLICT OF INTEREST STATEMENT

The authors declare no conflict of interest.

## ETHICS STATEMENT

Our study exclusively utilised publicly available videos, and we handled the data with strict confidentiality. No personal player information was accessed and therefore ethical permission was not required.

## Supporting information

Supplementary material ‐ ankle.

## Data Availability

Raw videos and data supporting the findings of this study are either publicly available and/or included in the supporting information.
