# Supplementary material for: Systematic video analysis of ankle sprain injuries in elite male football (soccer): Injury mechanisms, situational patterns, biomechanics and neurocognitive errors study: A study on 140 consecutive players
Source: Knee Surg Sports Traumatol Arthrosc. 2025 Sep 9;33(11):4035–49. doi: 10.1002/ksa.70049 (PMC12582226; doi:10.1002/ksa.70049)
Supplement: Supplementary file 1 — Supplementary material ‐ ankle. [file KSA-33-4035-s001.docx]

**Supplemental Material**

**Table S1.** QA-SIVAS scores for this study

| **Code** | **Present study** |
| --- | --- |
| **Objective stated** | ✓ |
| **Representative sample** | ✓ |
| **Information about sample** | ✓ |
| **Information about video source** | ✓ |
| **Applied methods** | ✓ |
| **Systematic approach to video analysis** | ✓ |
| **Medical report information** | × |
| **Background of raters** | ✓ |
| **Findings observed by more than one researcher** | ✓ |
| **A control group is included** | × |
| **Quantitative biomechanical analysis** | ✓ |
| **Main results** | ✓ |
| **Absolute numbers or proportions reported** | ✓ |
| **Details about injury context** | ✓ |
| **Example screenshots/video frames** | ✓ |
| **Findings are discussed within the context** | ✓ |
| **Clinical/practical implications** | ✓ |
| **Limitations** | ✓ |
| **Total Score** | 16 |

**Table S2.** Checklist for video evaluation

| **Variable** | **Category** |
| --- | --- |
| Playing phase before injury | Defensive/Offensive |
| Injury side | Right/Left |
| Dominant leg injured | Yes/No |
| Minute zone during the match | 0-5/5-10/10-15/15-20/20-25/25-30/30-35/35-40/40-45/45-50/50-55/  55-60/65-70/70-75/75-80/80-85/85-90/90+ |
| Minutes of effective gameplay | 0-5/5-10/10-15/15-20/20-25/25-30/30-35/35-40/40-45/45-50/50-55/  55-60/65-70/70-75/75-80/80-85/85-90/90+ |
| Field location at injury | - Defensive third/Midfield third/Offensive third  - Left side corridor/Middle corridor/Right side corridor |
| Player situational pattern  (only for indirect and noncontact injuries) | Pressing/Tackling/Being tackled/Landing from a jump/Regaining balance after kicking/Sliding/Offensive change of direction/ ball kicking/ Other |
| Player contact preceding injury | Yes/No |
| If contact, where? | Injured ankle/Foot of injured side/Tibia of injured side/ Injured leg/Pelvis/ Upper body/Uninjured leg |
| Injury classification | Direct contact/Indirect contact/Noncontact |
| Ankle biomechanics | Inversion/Eversion/High ankle/ Unsure |
| Leg loading at IF | Injured Leg/Uninjured Leg/None/Unsure |
| Horizontal speed | Zero/Low/High |
| Vertical speed | Zero/Low/High |

IF: injury frame.

**Table S3.** Checklist for biomechanical evaluation

| **Variable** | **Evaluation** |
| --- | --- |
| Trunk flexion (+ flexion, – extension) | Estimation to nearest 5° |
| Hip flexion (+ flexion, – extension) | Estimation to nearest 5° |
| Knee flexion (+ flexion, – extension) | Estimation to nearest 5° |
| Ankle flexion (+ dorsiflexion, – plantarflexion) | Estimation to nearest 5° |
| Foot strike | Heel/flat/toe/unsure |
| Trunk tilt (+ ipsilateral, – contralateral) | Estimation to nearest 5° |
| Trunk tilt | Toward injured leg/neutral/towards uninjured leg/unsure |
| Trunk rotation | Toward injured leg/neutral/towards uninjured leg/unsure |
| Frontal plane hip alignment | Abducted/neutral/adducted/unsure |
| Frontal plane knee alignment | Valgus/neutral/varus/unsure |
| Ankle tilt (+inversion, -eversion) |  |
| Foot position | Externally rotated/neutral/internally rotated |

IC: initial contact; IF: injury frame; IR: internal rotation; ADD: adduction.

**Table S4.** Number of ankle sprain injuries and rate of ankle sprain injuries per square meter according to divisions along the length and width of the soccer field (N = 140 injuries)

|  | **Length of the Field** | | |  | **Width of the Field** | | |
| --- | --- | --- | --- | --- | --- | --- | --- |
|  | **Defensive Third** | **Midfield Third** | **Offensive Third** |  | **Left Corridor** | **Middle Corridor** | **Right Corridor** |
| Square meters | 2450 | 2450 | 2450 |  | 1575 | 4200 | 1575 |
| No. of ankle sprain injuries | 43 | 50 | 47 |  | 22 | 87 | 31 |
| No. of ankle sprain injuries/m² | 0.018 | 0.020 | 0.019 |  | 0.014 | 0.021 | 0.020 |

**Table S5.** Number of ankle sprain injuries and rate of ankle sprain injuries per square meter according to the 11 different field zones (N = 140 Injuries)

|  | **Field Zone** | | | | | | | | | | | |
| --- | --- | --- | --- | --- | --- | --- | --- | --- | --- | --- | --- | --- |
|  | **1** | **2** | **3** | **4** | **5** | **6** | **7** | **8** | **9** | **10** | **11** |  |
| Square meters | 660 | 740 | 1400 | 740 | 660 | 525 | 525 | 525 | 525 | 525 | 525 |  |
| No. of ankle sprain injuries | 17 | 18 | 24 | 15 | 13 | 2 | 11 | 9 | 6 | 15 | 10 |  |
| No. of ankle sprain injuries/m² | 0.030 | 0.024 | 0.019 | 0.020 | 0.020 | 0.008 | 0.021 | 0.017 | 0.013 | 0.032 | 0.019 |  |

**Table S6.** Injuries according to league and/or cup competition (N =140).

| **Competition** | **Number** |
| --- | --- |
| **Domestic league** |  |
| Bundesliga | 27 |
| Premier League | 25 |
| Seria A | 25 |
| La Liga | 18 |
| Ligue 1 | 11 |
| **Domestic Cup** |  |
| DFB-Pokal (German Cup) | 4 |
| English Football League/ Caraboa Cup | 4 |
| Football Association (FA) Cup | 2 |
| Copa del Rey | 2 |
| Coupe de France | 2 |
| Coppa Italia | 1 |
| **European Cup** |  |
| Champions League | 4 |
| Europa League | 4 |
| Emirates Cup | 1 |
| **International matches** |  |
| UEFA Nations League | 3 |
| UEFA European Football Championship (U21s) | 2 |
| UEFA European Football Championship | 1 |
| **Friendly matches** |  |
| International team friendlies | 3 |
| Club International Friendlies | 1 |
